# Early warning score independently predicts adverse outcome and mortality in patients with acute pancreatitis

**DOI:** 10.1007/s00423-017-1581-x

**Published:** 2017-04-22

**Authors:** Michael J. Jones, Christopher P. Neal, Wee Sing Ngu, Ashley R. Dennison, Giuseppe Garcea

**Affiliations:** 1 Department of Hepatobiliary Surgery, University Hospitals of Leicester, Leicester, LE5 4PW UK; 2 Leicester General Hospital, Gwendolen Road, Leicester, LE5 4PW UK

**Keywords:** Acute pancreatitis, EWS, Outcome, Mortality, Scoring

## Abstract

**Purpose:**

The aim of this study was to compare the prognostic value of established scoring systems with early warning scores in a large cohort of patients with acute pancreatitis.

**Methods:**

In patients presenting with acute pancreatitis, age, sex, American Society of Anaesthesiologists (ASA) grade, Modified Glasgow Score, Ranson criteria, APACHE II scores and early warning score (EWS) were recorded for the first 72 h following admission. These variables were compared between survivors and non-survivors, between patients with mild/moderate and severe pancreatitis (based on the 2012 Atlanta Classification) and between patients with a favourable or adverse outcome.

**Results:**

A total of 629 patients were identified. EWS was the best predictor of adverse outcome amongst all of the assessed variables (area under curve (AUC) values 0.81, 0.84 and 0.83 for days 1, 2 and 3, respectively) and was the most accurate predictor of mortality on both days 2 and 3 (AUC values of 0.88 and 0.89, respectively). Multivariable analysis revealed that an EWS ≥2 was independently associated with severity of pancreatitis, adverse outcome and mortality.

**Conclusion:**

This study confirms the usefulness of EWS in predicting the outcome of acute pancreatitis. It should become the mainstay of risk stratification in patients with acute pancreatitis.

## Introduction

Acute pancreatitis has an associated mortality of approximately 6 % [1], and patients with persistent organ failure have a reported mortality of 36–50 % [1–3]. Fortunately, an increase in incidence has not been mirrored by an increase in mortality [4]. The revised Atlanta Classification System defines severe acute pancreatitis (SAP) as comprising of organ dysfunction lasting more than 48 h [5], which is associated with an increase in mortality [2, 3]. UK guidelines advocate early severity stratification, aggressive fluid resuscitation and prompt treatment of the underlying aetiology [6]. High dependency unit monitoring in all patients with SAP is recommended.

A multitude of severity scores for acute pancreatitis are currently in use. Early and accurate prediction of prognosis enables patients with or at risk of developing SAP to be identified and closely supported with intensive monitoring. Current scoring systems assess a combination of physiological, biochemical and/or imaging features. The suggested prognostic factors in UK guidelines include the Modified Glasgow Criteria (MGC) and the Acute Physiology and Chronic Health Evaluation (APACHE) II score [6]. Other validated scoring systems include the Ranson criteria and the Balthazar score.

Previous data have suggested that the early warning score (EWS) may be useful in screening patients to predict the severity of an episode of acute pancreatitis and for monitoring the response to treatment [7–9]. EWS is a bedside score that measures the following six values: heart rate, respiratory rate, conscious state, temperature, urine output and blood pressure (Table 1), and it is simple to calculate and hence readily available. It is purely clinical and required the addition of no biochemical or radiological tests. Regional variations in the scoring proforma exist within the UK, but the scores are generally homogenous. EWS reflects the systemic inflammatory response syndrome (SIRS), which is the main cause of organ dysfunction and mortality in many conditions including acute pancreatitis. The use of the EWS has been recognised in other critical surgical [10, 11] and medical conditions [12]. As well as its use in predicting prognosis, it facilitates a logical policy to guide the escalation of care (a high score will demand senior trainee or consultant review), meaning that it is invaluable in the clinical setting.

**Table 1:** University Hospitals of Leicester early warning score

Variable	Score^a^													
	3		2		1		0		1		2		3	
Heart rate			<40		40–50		51–100		101–110		111–129		>130	
Respiratory rate			≤8				9–14		15–20		21–29		≥30	
Temperature			<35.0		35.1–36.0		36.1–37.9		38.0–38.4		≥38.5			
CNS							Alert		Voice		Pain		Unconscious	
Urine (catheter)	Nil		<0.5 ml/kg for >2 h		<0.5 ml/kg for >1 h				>3 ml/kg for >2 h					
Urine (no catheter)	PU in 12 h, no						PU in 12 h, yes							
BP	Patient’s normal systolic (mmHg)													
Current systolic (mmHg)		200	190	180	170	160	150	140	130	120	110	100	90	80
	200	0	0	0	1	1	1	2	2	3	3	4	5	5
	190	0	0	0	0	1	1	1	2	2	3	3	4	5
	180	0	0	0	0	0	0	1	1	2	2	3	3	4
	170	1	1	0	0	0	0	1	1	2	2	3	3	4
	160	1	1	1	0	0	0	0	0	1	1	2	2	3
	150	1	1	1	1	0	0	0	0	0	1	1	2	2
	140	2	2	1	1	1	1	0	0	0	0	1	1	2
	130	2	2	2	1	1	1	0	0	0	0	0	1	1
	120	2	2	2	2	1	1	0	0	0	0	0	0	1
	110	3	3	2	2	2	2	1	0	0	0	0	0	0
	100	3	3	3	3	2	2	1	1	0	0	0	0	0
	90	4	4	3	3	3	3	2	2	1	0	0	0	0
	80	4	4	4	4	3	3	3	2	2	1	1	0	0
	70	4	4	4	4	4	4	3	3	2	2	2	1	0
	60	4	4	4	4	4	4	4	4	3	3	3	2	1
	50	5	5	5	5	5	5	5	5	4	4	4	3	2
	40	6	6	6	6	6	6	6	6	5	5	5	4	3

*CNS* central nervous system, *PU* passed urine, *BP* blood pressure

^a^Overall score is the sum of each individual variable score

Various isolated biochemical values have also been identified as potential markers of the severity of an episode of pancreatitis and are included in a range of scoring systems. Using a cutoff value of 150 mg/l, C-reactive protein (CRP) has been shown to be useful at 48 h following admission [6]. Leucocyte count is often incorporated within scoring systems such as Ranson criteria, MGC and APACHE II. The use of neutrophil-lymphocyte ratio (NLR) has been described in other critical and cardiac illnesses [13] and in some studies has recently been found to be useful in determining prognosis in patients with acute pancreatitis [14]. Azab et al. have studied the NLR in acute pancreatitis and demonstrated its usefulness in predicting rates of admission to intensive therapy unit (ITU) and prolonged lengths of stay [15].

This study re-examines the efficacy of EWS in determining the outcome of acute pancreatitis in the largest patient cohort reported to date. EWS was compared to other prognostic scores (including APACHE II, MGC and Ranson criteria) as well as haematological variables such as CRP, NLR and leucocyte count.

## Materials and methods

**Fig. 1:**
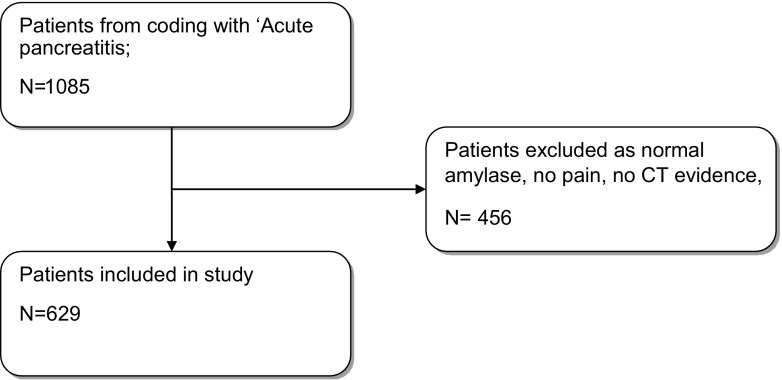
Patient selection

A retrospective observational study was undertaken. Patients admitted with a coding diagnosis of acute pancreatitis from 2007 to 2011 were identified from computerised records. Diagnostic criteria for acute pancreatitis were a serum amylase three times the upper limit of normal in patients with upper abdominal pain or radiological evidence of acute pancreatic inflammation; patients not meeting this criteria were excluded (Fig. 1). Patient age, sex, aetiology of pancreatitis, American Society of Anaesthesiologists (ASA) grade, MGC and Ranson criteria were recorded. Where applicable, the Balthazar computed tomography (CT) score was noted from the first CT scan available after index admission. APACHE II scores and EWS were collected during the first 3 days of admission to hospital. The worst values within a 24-h period were recorded for each physiological scoring system. The number of patients developing SIRS was also noted on days 1 to 3. The presence of SIRS was defined as any two of the following: temperature greater than 38 °C or less than 36 °C, heart rate greater than 90 beats per minute, respiratory rate greater than 20/min, PCO_2_ of less than 32 mm Hg and white blood cell counts greater than 12,000 or less than 4000 cells/mm^3^. The following biochemical and haematological parameters were also noted on days 1, 2 and 3: CRP, leucocyte count, neutrophil count, lymphocyte count and NLR.

These variables were compared between survivors and non-survivors, between patients with acute mild/moderate or severe pancreatitis and between patients with a favourable or adverse outcome. A favourable outcome was defined as survival of the episode of pancreatitis without high dependency unit (HDU) or ITU admission or operative intervention. An adverse outcome was defined as non-survival, admission to HDU or the need for operative intervention, excluding cholecystectomy.

### Statistical method

The Shapiro-Wilk test of normality was used to determine if the continuous variables were parametric or non-parametric. Parametric data was compared using the two-tailed *t* test and non-parametric data with the Mann-Whitney *U* test. Categorical data was compared using the Pearson’s chi-squared and Fisher’s exact tests as appropriate. Subsequently, receiver-operating characteristic (ROC) analysis was applied as a measure of the overall accuracy of individual markers. Univariable and multivariable binary logistic regression analyses were performed to identify variables independently associated with severity, adverse outcome and survival. All variables with a *p* value <0.10 on univariable analysis were taken forward into multivariable analysis, which was performed using a stepwise backward model. Continuous variables were analysed following paramedian split in these analyses. Analyses were conducted using SPSS version 20.0, and all *p* values were two sided. Significance was set at a *p* value of <0.05.

## Results

A total of 629 patients were identified and fulfilled the admission criteria for the study. Three hundred and nine (49.1 %) were male and 320 (50.9 %) were female. There were 55 deaths, including 4 operative cases, resulting in a mortality rate of 8.7 %. Fourteen surviving patients required surgical intervention. HDU or ITU admission was required in 62 cases, with 22 of these patients dying, giving a mortality rate following HDU/ITU admission of 35.5 %. In total, 101 patients had an adverse outcome. The cause of pancreatitis was gallstones in 343 cases (54.5 %), with the remaining cases being caused by alcohol (14 %), ERCP (5.2 %), other uncommon causes (4.1 %) and unknown cause (22.1 %). There was insufficient data relating to CT grading of pancreatitis severity for this to be included in the analysis.

### Comparison of variables between groups

Patient age and ASA grade were significantly higher in non-survivors, severe episodes and in episodes with an adverse outcome (Table 2). The remaining prognostic scoring systems evaluated all demonstrated significantly higher values for all three outcomes (*p* < 0.001).

**Table 2:** Clinicopathological and laboratory data in patients with acute pancreatitis compared according to severity, adverse outcome and mortality

		Severity			Adverse outcome			Mortality		
		Mild/moderate	Severe	*p* value	Non-adverse	Adverse	*p* value	Survivors	Non-survivors	*p* value
Age		58 (15–102)	67 (20–98)	<0.001	59 (15–102)	70 (24–93)	<0.001	56.96 (±20.19)	74.65 (±13.52)	<0.001
Age	≥65	287/191	66/85	<0.001	315/213	38/63	<0.001	342/232	11/44	<0.001
Sex	Male	251	58	0.003	260	49	0.893	292	28	0.996
	Female	227	93	0.003	268	52	0.893	282	27	0.996
Aetiology	Gallstones	269	74	0.118	301	42	0.004	321	22	0.023
	EtOH	67	21	0.973	77	11	0.327	83	5	0.273
	ERCP	26	7	0.699	26	7	0.407	29	4	0.480
	Other	21	5	0.560	24	2	0.409	115	24	<0.001
	Unknown	95	44	0.017	100	39	<0.001	26	0	0.155
ASA grade		6 (0–18)	12 (0–31)	<0.001	2 (1–4)	3 (1–5)	<0.001	2 (1–4)	3 (1–5)	<0.001
		5 (0–18)	9 (0–40)	<0.001	428/100	100/56	<0.001	457/117	16/39	<0.001
		4 (0–22)	8 (0–40)	<0.001	519/9	94/7	0.007	563/11	50/5	0.009
MCG		1 (0–6)	2 (0–7)	<0.001	1 (0–5)	3 (0–7)	<0.001	1 (0–7)	3 (0–6)	<0.001
Ranson		1 (0–5)	2 (0–6)	<0.001	1 (0–5)	2 (0–6)	<0.001	1 (0–6)	3 (0–6)	<0.001
Apache II	Day 1	6 (0–18)	12 (0–31)	<0.001	7 (0–20)	12 (0–31)	<0.001	7 (0–30)	13.5 (3–31)	<0.001
	Day 2	5 (0–18)	9 (0–40)	<0.001	5 (0–22)	10 (0–40)	<0.001	5 (0–30)	12 (3–40)	<0.001
	Day 3	4 (0–22)	8 (0–40)	<0.001	5 (0–22)	9 (0–40)	<0.001	5 (0–34)	10 (1–40)	<0.001
SIRS	Day 1	102	63	<0.001	112	53	<0.001	135	30	<0.001
	Day 2	82	57	<0.001	91	48	<0.001	113	26	<0.001
	Day 3	72	46	<0.001	75	43	<0.001	95	23	<0.001
EWS	Day 1	1 (0–9)	2 (0–12)	<0.001	1 (0–9)	4 (0–12)	<0.001	1 (0–12)	4 (1–11)	<0.001
	Day 2	1 (0–9)	4 (0–12)	<0.001	1 (0–8)	5 (0–12)	<0.001	1 (0–12)	5 (1–12)	<0.001
	Day 3	1 (0–10)	2.5 (0–10)	<0.001	1 (0–10)	4 (0–10)	<0.001	1 (0–10)	5 (0–10)	<0.001
CRP	Day 1	12 (3–675)	32 (3–352)	<0.001	13 (3–675)	74.5 (3–382)	<0.001	14 (3–675)	39 (3–357)	0.002
	Day 2	89 (3–343)	154.5 (11–411)	0.004	91.5 (3–411)	188 (3–393)	0.001	96 (3–411)	168.5 (37–352)	0.028
	Day 3	130 (3–410)	279.5 (3–433)	<0.001	132 (3–410)	274.5 (3–433)	0.019	143.5 (3–433)	282.5 (196–290)	0.050
Leucocyte count	Day 1	12.5 (2.6–34.9)	15.0 (3.5–29.5)	<0.001	12.7 (2.6–34.9)	15.5 (3.5–29.5)	<0.001	12.8 (2.6–34.9)	14.4 (3.5–25.5)	0.016
	Day 2	11.1 (3.2–35.9)	12.0 (0.7–29.8)	0.117	11.25 (2.10–35.9)	11.85 (0.7–30.3)	0.256	11.3 (2.1–35.9)	12.4 (0.7–30.3)	0.203
	Day 3	10.2 (3.3–27.9)	10.85 (2.60–41.7)	0.308	10.2 (2.6–41.7)	10.9 (3.8–25.4)	0.184	10.2 (2.6–41.7)	12.1 (3.8–25.4)	0.208
Neutrophil count	Day 1	10.5 (2.12–33.47)	12.60 (0.42–28.32)	0.002	10.66 (0.42–33.47)	12.83 (3.16–28.32)	<0.001	10.8 (0.42–33.47)	12.76 (3.16–23.57)	0.006
	Day 2	9.00 (0.96–33.98)	9.89 (0.36–24.90)	0.096	9.10 (0.36–33.98)	10.05 (0.47–28.53)	0.073	9.1 (0.36–33.98)	10.92 (0.47–28.53)	0.038
	Day 3	7.70 (2.10–25.16)	9.21 (0.57–22.59)	0.134	7.56 (0.57–25.16)	9.71 (1.01–22.59)	0.054	7.73 (0.57–25.16)	9.83 (1.01–22.59)	0.129
Lymphocyte count	Day 1	1.16 (0.20–4.71)	1.12 (0.13–21.70)	0.878	1.20 (0.13–21.70)	0.99 (0.24–3.18)	0.188	1.20 (0.13–21.70)	0.92 (0.24–3.18)	0.017
	Day 2	1.21 (0.33–4.43)	0.92 (0.15–11.17)	<0.001	1.21 (0.15–11.17)	0.80 (0.17–3.99)	<0.001	1.19 (0.15–11.17)	0.70 (0.17–3.99)	<0.001
	Day 3	1.28 (0.35–4.98)	0.94 (0.20–6.70)	<0.001	1.27 (0.26–4.07)	0.82 (0.20–6.70)	<0.001	1.23 (0.26–4.07)	0.78 (0.20–6.70)	<0.001
NLR	Day 1	9.70 (0.95–78.50)	10.48 (0.02–47.31)	0.139	9.47 (0.02–78.50)	11.39 (2.16–45.79)	0.004	9.6 (0.02–78.5)	12.3 (2.2–45.8)	0.001
	Day 2	7.84 (0.48–58.22)	11.30 (0.03–74.00)	<0.001	7.86 (0.03–74.00)	12.17 (1.83–58.33)	<0.001	8.0 (0.3–74.0)	14.2 (1.8–58.3)	<0.001
	Day 3	6.22 (0.69–40.68)	10.20 (0.14–31.15)	<0.001	6.30 (0.14–40.68)	11.66 (1.00–30.00)	<0.001	6.7 (0.14–40.7)	13.5 (1.0–28.7)	<0.001

All values are median values with range in parentheses unless otherwise indicated

*EtOH* alcohol, *ERCP* endoscopic retrograde cholangiopancreatography, *ASA* American Society of Anaesthesiologists, *MGC* Modified Glasgow Criteria, *Ranson* Ranson criteria, *APACHE II* Acute Physiology and Chronic Health Evaluation, *SIRS* systemic inflammatory response syndrome, *EWS* early warning score, *CRP* C-reactive protein, *NLR* neutrophil/lymphocyte ratio

Total leucocyte count only demonstrated a significant association with mortality, severity and adverse outcome on day 1 with no significant association between day 2 or 3 leucocyte count and any outcome. When NLR was compared between groups, significantly higher NLR was found in severe cases on days 2 and 3 and in non-survivors and in patients with an adverse outcome on all 3 days. Regarding its components, neutrophil count was significantly higher in severe cases and patients with adverse outcome on day 1 only and in non-survivors on days 1 and 2. By contrast, lymphocyte count was significantly lower in all three groups on days 2 and 3 and was also significantly lower in non-survivors on day 1. Day 3 neutrophil count was not significantly different between any of the groups.

### ROC analysis

Comparison of area under curve (AUC) data from the ROC analysis for clinical prognostic scoring systems revealed that EWS, although highly statistically significant on all 3 days (*p* < 0.001), was inferior in predicting severity of acute pancreatitis when compared to the MGC and APACHE II on day 1 (AUC values 0.71 vs. 0.78 and 0.76, respectively; Table 3). Its predictive value on days 2 and 3 (AUC values 0.75 and 0.70) was marginally lower than those of APACHE II. EWS demonstrated the most accurate predictive ability for adverse outcome and was also the best predictor of adverse outcome amongst all of the clinical and laboratory variables assessed (AUC values 0.81, 0.84 and 0.83 for days 1, 2 and 3, respectively). Although EWS was inferior in predictive value to APACHE II on day 1 (AUC values 0.83 vs. 0.84, respectively), it most accurately predicted mortality on both day 2 and day 3 amongst all variables assessed (AUC values 0.88 and 0.89, respectively).

**Table 3:** The AUC predictive value of clinical and laboratory variables in predicting severity, outcome and mortality associated with acute pancreatitis

		Severity				Adverse outcome				Mortality			
		AUC	SE	95 % CI	*p* value	AUC	SE	95 % CI	*p* value	AUC	SE	95 % CI	*p* value
Age		0.62	0.03	0.57–0.67	<0.001	0.64	0.03	0.58–0.70	<0.001	0.76	0.03	0.70–0.82	<0.001
ASA grade		0.64	0.03	0.59–0.69	<0.001	0.71	0.03	0.66–0.77	<0.001	0.79	0.03	0.74–0.851	<0.001
Balthazar		0.58	0.04	0.50–0.65	0.059	0.60	0.04	0.52–0.69	0.023	0.61	0.06	0.49–0.72	0.071
MGC		0.78	0.02	0.73–0.82	<0.001	0.80	0.03	0.74–0.85	<0.001	0.79	0.04	0.72–0.86	<0.001
Ranson		0.73	0.03	0.68–0.78	<0.001	0.69	0.03	0.62–0.75	<0.001	0.75	0.04	0.67–0.83	<0.001
APACHE II	Day 1	0.76	0.02	0.72–0.81	<0.001	0.77	0.03	0.72–0.82	<0.001	0.84	0.03	0.79–0.89	<0.001
	Day 2	0.75	0.03	0.71–0.80	<0.001	0.81	0.02	0.76–0.86	<0.001	0.85	0.03	0.80–0.90	<0.001
	Day 3	0.73	0.03	0.68–0.78	<0.001	0.79	0.03	0.74–0.84	<0.001	0.80	0.03	0.74–0.86	<0.001
SIRS	Day 1	0.63	0.03	0.57–0.68	<0.001	0.69	0.03	0.63–0.75	<0.001	0.71	0.04	0.62–0.79	<0.001
	Day 2	0.65	0.03	0.60–0.71	<0.001	0.72	0.03	0.66–0.79	<0.001	0.73	0.05	0.64–0.81	<0.001
	Day 3	0.62	0.03	0.56–0.68	<0.001	0.71	0.04	0.64–0.78	<0.001	0.71	0.05	0.61–0.80	<0.001
EWS	Day 1	0.71	0.03	0.66–0.77	<0.001	0.81	0.03	0.75–0.86	<0.001	0.83	0.03	0.77–0.88	<0.001
	Day 2	0.75	0.03	0.70–0.81	<0.001	0.84	0.03	0.78–0.90	<0.001	0.88	0.03	0.83–0.93	<0.001
	Day 3	0.70	0.03	0.64–0.76	<0.001	0.83	0.04	0.76–0.90	<0.001	0.89	0.03	0.83–0.96	<0.001
CRP	Day 1	0.64	0.03	0.57–0.70	<0.001	0.70	0.04	0.62–0.79	<0.001	0.66	0.05	0.56–0.75	0.002
	Day 2	0.66	0.05	0.56–0.76	0.004	0.70	0.06	0.58–0.82	0.001	0.68	0.07	0.55–0.81	0.028
	Day 3	0.71	0.05	0.61–0.81	<0.001	0.67	0.06	0.55–0.79	0.019	0.74	0.05	0.65–0.83	0.050
Leucocyte count	Day 1	0.61	0.03	0.56–0.67	<0.001	0.63	0.03	0.56–0.69	<0.001	0.60	0.04	0.52–0.69	0.016
	Day 2	0.55	0.03	0.49–0.62	0.117	0.54	0.04	0.47–0.61	0.256	0.56	0.05	0.46–0.66	0.203
	Day 3	0.54	0.04	0.47–0.60	0.308	0.55	0.04	0.48–0.63	0.184	0.57	0.05	0.47–0.66	0.208
Neutrophil count	Day 1	0.59	0.03	0.53–0.64	0.002	0.62	0.03	0.56–0.69	<0.001	0.62	0.04	0.54–0.70	0.006
	Day 2	0.56	0.03	0.49–0.62	0.096	0.57	0.04	0.49–0.64	0.073	0.60	0.05	0.51–0.69	0.038
	Day 3	0.55	0.04	0.48–0.62	0.134	0.58	0.04	0.51–0.65	0.054	0.58	0.05	0.49–0.67	0.129
Lymphocyte count	Day 1	0.50	0.03	0.45–0.56	0.878	0.54	0.03	0.48–0.61	0.188	0.60	0.04	0.52–0.68	0.017
	Day 2	0.67	0.03	0.61–0.74	<0.001	0.73	0.03	0.66–0.79	<0.001	0.79	0.04	0.71–0.86	<0.001
	Day 3	0.67	0.03	0.60–0.74	<0.001	0.72	0.04	0.64–0.79	<0.001	0.73	0.05	0.63–0.83	<0.001
NLR	Day 1	0.54	0.03	0.49–0.60	0.139	0.60	0.03	0.53–0.66	0.004	0.64	0.04	0.56–0.71	0.001
	Day 2	0.66	0.03	0.60–0.71	<0.001	0.69	0.03	0.62–0.76	<0.001	0.75	0.04	0.67–0.82	<0.001
	Day 3	0.65	0.03	0.58–0.71	<0.001	0.69	0.04	0.62–0.77	<0.001	0.70	0.05	0.61–0.79	<0.001

*ASA* American Society of Anaesthesiologists, *Balthazar* Balthazar CT severity index, *MGC* Modified Glasgow Criteria, *Ranson* Ranson criteria, *APACHE II* Acute Physiology and Chronic Health Evaluation, *SIRS* systemic inflammatory response syndrome, *EWS* early warning score, *CRP* C-reactive protein, *NLR* neutrophil/lymphocyte ratio, *AUC* area under the receiver-operating characteristic curve, *SE* standard error, *95 % CI* 95 % confidence interval

### Binary logistic regression analysis

Univariable logistic regression analysis demonstrated that all of the prognostic clinicopathological scoring systems assessed (ASA, MGC, APACHE II, SIRS, EWS), measured on all 3 days, showed highly significant associations with severity of pancreatitis, adverse outcome and mortality (all *p* < 0.001; Table 4). In addition, patient age over 60 demonstrated a highly significant association with all three outcomes (all *p* < 0.001).

**Table 4:** Univariable binary logistic regression for factors associated with severity, adverse outcome and mortality

		Cutoff	Severity			Adverse outcome			Mortality		
			HR	95 % CI	*p* value	HR	95 % CI	*p* value	HR	95 % CI	*p* value
Age		≤60/>60	2.11	1.44–3.20	<0.001	2.75	1.72–4.39	<0.001	8.51	3.59–20.19	<0.001
Sex		M/F	0.56	0.39–0.82	0.003	0.97	0.63–1.49	0.893	1.00	0.57–1.74	0.996
ASA		1–2/3–4	1.65	1.36–2.01	<0.001	2.31	1.84–2.89	<0.001	3.09	2.27–4.20	<0.001
MGC		0–1/≥2	6.78	4.44–10.35	<0.001	8.86	5.11–15.37	<0.001	7.81	3.75–16.27	<0.001
Ranson		0–1/≥2	4.25	2.84–6.37	<0.001	3.16	1.99–5.03	<0.001	4.28	2.23–8.22	<0.001
Apache II	Day 1	0–6/≥7	6.65	4.05–10.93	<0.001	7.43	3.87–14.26	<0.001	21.89	5.27–90.94	<0.001
	Day 2	0–4/≥5	4.25	2.84–6.37	<0.001	3.16	1.99–5.03	<0.001	4.283	2.23–8.22	<0.001
	Day 3	0–4/≥5	3.33	2.15–5.15	<0.001	8.04	4.07–15.86	<0.001	9.99	3.54–28.16	<0.001
SIRS	Day 1	Y/N	3.14	2.09–4.73	<0.001	5.21	3.22–8.41	<0.001	5.82	3.04–11.15	<0.001
	Day 2		4.07	2.62–6.33	<0.001	7.26	4.28–12.34	<0.001	7.13	3.49–14.59	<0.001
	Day 3		3.28	2.07–5.19	<0.001	6.94	4.07–11.82	<0.001	6.33	3.33–12.77	<0.001
EWS	Day 1	0–1/≥2	3.63	2.32–5.68	<0.001	7.32	4.31–12.42	<0.001	16.82	5.14–55.01	<0.001
	Day 2		4.99	3.06–8.13	<0.001	9.956	4.65–21.33	<0.001	21.96	5.22–92.31	<0.001
	Day 3		3.56	2.22–5.69	<0.001	11.56	5.17–26.16	<0.001	39.36	5.29–292.61	<0.001
CRP	–	25	2.35	1.46–3.79	<0.001	3.32	1.77–6.22	<0.001	2.44	1.16–5.12	0.019
Leucocyte count	Day 1	Median (12.9)	2.24	1.52–3.31	<0.001	2.20	1.38–3.51	0.001	1.64	0.91–2.94	0.100
	Day 2	Median (11.4)	1.36	0.87–2.11	0.177	1.24	0.76–2.03	0.394	1.50	0.78–2.89	0.225
	Day 3	Median (10.4)	1.53	0.95–2.44	0.079	1.53	0.90–2.62	0.119	2.29	1.08–4.86	0.030
Neutrophil count	Day 1	Median (10.9)	1.95	1.32–2.88	0.001	2.20	1.36–3.55	0.001	1.77	0.98–3.19	0.060
	Day 2	Median (9.3)	1.53	0.97–2.40	0.068	1.37	0.81–2.29	0.239	1.65	0.85–3.19	0.140
	Day 3	Median (8.3)	1.54	0.96–2.48	0.073	2.06	1.17–3.63	0.013	2.62	1.21–5.66	0.014
Lymphocyte count	Day 1	Median (1.2)	0.87	0.61–1.30	0.534	0.60	0.38–0.96	0.033	0.40	0.22–0.75	0.004
	Day 2	Median (1.1)	0.35	0.21–0.56	<0.001	0.23	0.13–0.42	<0.001	0.15	0.06–0.36	<0.001
	Day 3	Median (1.2)	0.30	0.18–0.51	<0.001	0.21	0.11–0.41	<0.001	0.28	0.12–0.64	0.003
NLR	Day 1	Median (9.9)	1.37	0.94–2.01	0.105	2.47	1.52–4.02	<0.001	4.00	2.03–8.03	<0.001
	Day 2	Median (8.6)	2.73	1.70–4.39	<0.001	3.99	2.21–7.18	<0.001	5.64	2.44–13.07	<0.001
	Day 3	Median (7.2)	2.61	1.59–4.28	<0.001	3.91	2.09–7.31	<0.001	4.38	1.85–10.34	0.001

*ASA* American Society of Anaesthesiologists, *MGC* Modified Glasgow Criteria, *Ranson* Ranson criteria, *APACHE II* Acute Physiology and Chronic Health Evaluation, *SIRS* systemic inflammatory response syndrome, *EWS* early warning score, *CRP* C-reactive protein, *NLR* neutrophil/lymphocyte ratio, *HR* hazard ratio, *95 % CI* 95 % confidence interval

Amongst the haematological variables, high leucocyte and neutrophil counts on day 1 were significantly associated with severity and adverse outcome. A high leucocyte count on day 3 was significantly associated with mortality, whilst a high neutrophil count on day 3 was associated with an adverse outcome and mortality. Highly significant associations were noted between both lymphocyte count and NLR and all three outcomes on both day 2 (all *p* < 0.001) and day 3 (all *p* ≤ 0.003).

Multivariable analysis revealed that EWS and low lymphocyte count were the dominant factors independently associated with all three outcomes. EWS ≥2 (measured on all 3 days) was independently associated with severity of pancreatitis (Table 5). In addition, low day 2 and day 3 lymphocyte counts were independently associated with disease severity. EWS ≥2 also demonstrated an independent association with adverse outcome on all 3 days, whilst low day 2 and day 3 lymphocyte counts were also independently associated with severity. In relation to mortality, multivariable analysis demonstrated that EWS ≥2 was independently associated with death following pancreatitis when measured on day 1, day 2 or day 3. A low day 2 lymphocyte count was also independently associated with mortality.

**Table 5:** Multivariable binary logistic regression analyses for factors independently associated with severity, adverse outcome and mortality in patients with acute pancreatitis

Day	Variable	Group	Severity			Adverse outcome			Mortality		
			HR	95 % CI	*p* value	HR	95 % CI	*p* value	HR	95 % CI	*p* value
Day 1	ASA	I and II vs. III and IV	–	–	–	1.90	1.39–2.60	<0.001	3.55	2.28–5.55	<0.001
	EWS	0–1 vs. ≥2	5.14	2.47–10.69	<0.001	4.03	2.12–7.67	<0.001	5.41	2.23–13.14	<0.001
	Lymphocyte count	Above/below median	–	–	–	–	–	–	0.34	0.14–0.82	0.017
Day 2	Age	<60 vs. ≥60	–	–	–	–	–	–	5.85	1.19–28.7	0.029
	EWS	0–1 vs. ≥2	2.34	1.03–5.31	0.043	11.44	4.56–28.69	<0.001	7.76	1.59–37.98	0.011
	Lymphocyte count	Above/below median	0.46	0.22–0.95	0.036	0.37	0.16–0.86	0.021	0.16	0.04–0.77	0.023
Day 3	EWS	0–1 vs. ≥2	2.97	1.54–5.72	0.001	46.83	9.12–240.51	<0.001	42.48	5.36–337.25	<0.001
	Lymphocyte count	Above/below median	0.48	0.24–0.96	0.038	0.34	0.12–0.99	0.048	–	–	–

*ASA* American Society of Anaesthesiologists, *EWS* early warning score, *HR* hazard ratio, *95 % CI* 95 % confidence interval

Of note, when univariable and multivariable analyses were repeated to analyse continuous variables as (i) continuous data and (ii) following division around ROC-determined ‘optimal’ cut points, the same variables were found to have independent significance on multivariable analysis (data not shown).

## Discussion

In this study, early warning scores are independently predictive of an adverse outcome and mortality in patients with acute pancreatitis. Whilst EWS was marginally inferior to APACHE II in ROC severity prediction, it was superior in predicting adverse outcome and mortality. EWS also compared favourably with other validated clinicopathological scoring systems including the Modified Glasgow Criteria and Ranson criteria. EWS demonstrated independence when predicting severity prognosis, adverse outcome and mortality. The only other scoring systems identified by this study as showing independence was ASA grade (predicting adverse outcome and mortality) on day 1. Although a range of clinical and pathological data make up each scoring system, there are similarities between them. EWS consists of purely physiological data, and it is therefore interesting that it performed better than scoring systems reliant upon laboratory data. EWS also performed better than any single biochemical variable. EWS represents the acute inflammatory response, and this underscores the recognition that the severity of SIRS in acute pancreatitis is directly linked to an increased risk of an unfavourable outcome. The findings of this study confirm previous work, demonstrating that the EWS is a predictor of mortality [7].

The EWS can be calculated at the bedside allowing a prediction of likely outcome to be made almost immediately following a clinical review. Early risk prediction allows for an aggressive management to be commenced at an earlier stage. This study was performed using a centre-specific EWS (Table 1), and a score of 2 or more indicates a high risk of severity, adverse outcome and mortality. Centre-specific early warning scores are limited as data is not reproducible and hence comparable between centres [16]. A national EWS is currently being introduced and has already been validated as a predictor of cardiac arrest, ITU admission and mortality [17]. As this becomes more widespread, a standardised EWS cutoff can be used to predict prognosis and the consistency will make it applicable nationally.

EWS is a dynamic tool, easily repeatable at 15-min intervals that make it particularly valuable for the monitoring of disease progression and can thus be used to guide a clinician in appropriate management. Haemodynamic instability can be easily recognised and will prompt aggressive fluid resuscitation, whilst low oxygen saturation and tachypnoea will demand oxygen therapy and possibly further respiratory support. Acute pancreatitis is a disease that can be associated with a rapid change in clinical condition, and careful monitoring is needed to ensure that patients are managed in a timely fashion.

The APACHE II score has been utilised for the study of a range of conditions in ITU populations. It compares favourably with other scoring systems in the context of acute pancreatitis, although it has been found to be inaccurate when predicting the development of necrotising pancreatitis [18]. In this study, APACHE II correlated most accurately with mortality on ROC analysis, excluding day 3, and was relatively accurate with an AUC on day 2 of 0.85 (CI 0.80–0.90) and outperformed EWS in severity stratification. It did not, however, demonstrate any independent significance for any outcome assessed.

The Modified Glasgow Criteria and Ranson criteria also performed reasonably well with MGC outperforming Ranson criteria in severity, adverse outcome and mortality prediction. MGC demonstrated an AUC value of 0.80 (0.74–0.85) in relation to adverse outcome, which is better than previously demonstrated [7].

The bedside index of severity in acute pancreatitis (BISAP) has been examined by other studies looking at prognostic factors in acute pancreatitis [19, 20]. It combines age, SIRS, blood urea nitrogen, mental state and the presence of a pleural effusion. It has a reported area under the curve of 0.87 (*p* < 0.001) [19] in predicting severity and an AUC value of 0.86 in predicting death [20]. One study, however, demonstrated that multifactorial prognostic scores did not correlate well with outcome [21]. CRP and interleukin-6 have also been studied, and results suggest that they have a role in the prediction of severity [22]. Results from the present studies have demonstrated that CRP is useful and that its predictive value improved with time (AUC 0.64 to 0.71).

Whilst NLR and neutrophil count were significantly associated with outcome, the effect was not independent of clinicopathological scoring systems. Results did demonstrate that a low lymphocyte count had an independent prognostic significance for all three outcomes. Neutrophilia represents the same inflammatory process that is driving a rise in EWS, and given that the prognostic value of EWS is so strong may explain why neutrophil count and NLR lacked independent prognostic value on multivariable analysis. In contrast, the immunosuppressive effect that results in a falling lymphocyte count is a different process with prognostic significance, which is independent of EWS and other clinicopathological scoring systems [23]. As previously noted by Suppiah et al., further work is needed to assess the significance of lymphopenia in SAP [14].

The retrospective nature of this study meant that data collection was incomplete for some data points. Relatively few numbers of patients underwent CT scanning, and consequently, this yielded too small a cohort to enable meaningful analysis of Balthazar score. As with the majority of studies looking at pancreatitis severity, most data collection commenced following admission to hospital as opposed to the onset of pain. This is a fundamental source of inaccuracy for all studies examining prognostic variables in acute pancreatitis, but it is difficult to see how this can be avoided.

Early severity stratification remains a mainstay of the management of patients with SAP. In this study, lymphocyte count has also demonstrated its usefulness, and further work is needed to see if it is possible to incorporate it into existing scoring systems. This work further highlights the consistent value of EWS in risk prediction and monitoring of patients with SAP, and its use is advocated in all patients presenting with acute pancreatitis.
